# Mortality related to Verona Integron-encoded Metallo-β-lactamase-positive *Pseudomonas aeruginosa*: assessment by a novel clinical tool

**DOI:** 10.1186/s13756-019-0556-9

**Published:** 2019-06-19

**Authors:** Marjolein C. Persoon, Anne F. Voor in ‘t holt, Maurits P. A. van Meer, Karen C. Bokhoven, Diederik Gommers, Margreet C. Vos, Juliëtte A. Severin

**Affiliations:** 1 Department of Medical Microbiology and Infectious Diseases, Erasmus MC University Medical Center Rotterdam, Dr. Molewaterplein 40, 3015 GD Rotterdam, The Netherlands; 2 Department of Adult Intensive Care, Erasmus MC University Medical Center Rotterdam, Doctor Molewaterplein, 40 3015 GD Rotterdam, The Netherlands

**Keywords:** *Pseudomonas aeruginosa*, Mortality, Carbapenem, Microbial drug resistance, Infection control, Intensive care units

## Abstract

**Background:**

Verona Integron-encoded Metallo-β-lactamase-positive *Pseudomonas aeruginosa* (VIM-PA) can cause nosocomial infections and may be responsible for increased mortality. Multidrug resistance in VIM-PA complicates treatment. We aimed to assess the contribution of VIM-PA to mortality in patients in a large tertiary care hospital in the Netherlands.

**Methods:**

A focus group of five members created a scheme to define related mortality based on clinical and diagnostic findings. Contribution to mortality was categorized as “definitely”, “probably”, “possibly”, or “not” related to infection with VIM-PA, or as “unknown”. Patients were included when infected with or carrier of VIM-PA between January 2008 and January 2016. Patient-related data and specific data on VIM-PA cultures were retrieved from the electronic laboratory information system. For patients who died in our hospital, medical records were independently reviewed and thereafter discussed by three physicians.

**Results:**

A total of 198 patients with any positive culture with VIM-PA were identified, of whom 95 (48.0%) died. Sixty-seven patients died in our hospital and could be included in the analysis. The death of 15 patients (22.4%) was judged by all reviewers to be definitely related to infection with VIM-PA. In 17 additional patients (25.4%), death was probably or possibly related to an infection with VIM-PA. The level of agreement was 65.7% after the first evaluation, and 98.5% after one session of discussion.

**Conclusion:**

Using our assessment tool, infections with VIM-PA were shown to have an important influence on mortality in our complex and severely ill patients. The tool may be used for other (resistant) bacteria as well but this needs further exploration.

**Electronic supplementary material:**

The online version of this article (10.1186/s13756-019-0556-9) contains supplementary material, which is available to authorized users.

## Background

*Pseudomonas aeruginosa* infections are associated with substantial morbidity and mortality in hospitalised and immunocompromised patients [1–3]. Carbapenem resistance in *P. aeruginosa* isolates is an emerging problem worldwide, and also in Dutch hospitals outbreaks have occurred [4–8]. Besides changes in outer membrane porins and increased efflux pump activity, carbapenem resistance in *P. aeruginosa* can be caused by presence of carbapenemase enzymes, of which the metallo-β-lactamases (MBLs) are the most widespread. In the Netherlands, the imipenemase (IMP) and Verona Integron-encoded MBL (VIM) are the most common MBLs in *P. aeruginosa* [4]. Studies on various Gram-negative bacteria suggest that resistance to carbapenem antibiotics may be responsible for increased mortality [9, 10]. On the other hand, Carmeli et al. and Suarez et al. did not demonstrate an association between baseline resistance to any of four antipseudomonal antibiotics tested and adverse outcomes of patients with a clinical culture positive for *P. aeruginosa* [11]. Emergence of resistance, however, was associated with a significantly increased number of adverse outcomes [11]. The same study group conducted a prospective multicentre study in which carbapenem resistance was associated with significantly increased mortality rates, but the impact on mortality was higher for less severely ill patients [12]. Still, data about the attributable mortality of infection with VIM-positive *P. aeruginosa* (VIM-PA) are lacking. Furthermore, it is challenging to define and subsequently determine the attributable mortality due to infection with VIM-PA, since immune status and host factors play important roles*.* To assess the effect of infection with a bacterium on mortality, large numbers of patients must be included to reach statistical significance. For VIM-PA in the Netherlands, these large numbers of inclusion cannot be reached due to low overall prevalence. Therefore, different research strategies should be explored to gain more insight into the effects of VIM-PA on mortality.

Since 2003, VIM-PA is present in the Erasmus MC University Medical Center Rotterdam (Erasmus MC), The Netherlands [4]. Multiple colonisations and infections occurred and extensive infection prevention measures were taken [4, 8]. However, the impact on clinical outcome in our patients was not systematically studied previously. In the current study, we aimed to assess the contribution of VIM-PA to mortality. For this purpose, we developed a clinical tool and retrospectively analysed medical records of all deceased patients with any positive culture with VIM-PA.

## Methods

### Setting

The study was performed in the Erasmus MC, a 1200-bed tertiary-care hospital which has three high-level adult intensive care (ICU) wards.

### Assessment tool

A focus group of five members (two clinical microbiologists, two residents in clinical microbiology and a clinical epidemiologist) made a scheme of definitions based on possible clinical and diagnostic findings, in order to determine whether death of a patient with any positive VIM-PA culture could be related to infection with VIM-PA. This was done before patient data of included patients were reviewed and was based on clinical experience of the four medical doctors in the focus group, in combination with generally accepted definitions of sepsis described in Additional file 1 [13].

Culture results and sepsis criteria were included by the focus group because this information was judged to be crucial in defining related mortality and it could be obtained fairly objectively. However, cultures are not always taken for unknown reasons and the used sepsis criteria from literature are not applicable in every patient (e.g. in ICU patients who receive medication for support of vital functions). Furthermore, in complex patients it may be difficult to interpret clincical findings described by the patients physician and not all clinically relevant findings may be documented. Therefore, four categories were defined which represent a decreasing probablility that the VIM-PA had contributed to mortality. A fifth category represented cases in which too little data was available for a meaningful assessment. In summary, contribution to mortality was categorized as (i) definitely related, (ii) probably related, (iii) possibly related, (iv) not related to infection with VIM-PA, or as (v) unknown (Table 1). The categories were developed during four meetings with discussions and were pilot-tested with data of a few patients before use.

**Table 1:** Definitions of categories for the assessment of mortality related to Verona Integron-encoded Metallo-β-lactamase positive *Pseudomonas aeruginosa*

Definition	Description
Definitely related	A patient died due to sepsis, severe sepsis or septic shock and had a recent (< 10 days) blood culture with VIM-PA, without other pathogens in a blood culture.*
Probably related	A patient died due to sepsis, without a recent positive blood culture (blood cultures were not taken or negative < 10 days of death), but with a nosocomial infection according to CDC definitions^1^ AND the strong suspicion of VIM-PA as the pathogen causing sepsis (cultures from sterile sites e.g.; ascites, abscess, bile, empyema are positive with VIM-PA). OR A patient died due to sepsis and had a positive blood culture with VIM-PA longer than 10 days before death but within one month of death, without another pathogen isolated in blood cultures.
Possibly related	A patient died due to sepsis and there were cultures with VIM-PA in sterile specimens other than blood cultures within two months prior to death, with no other cultured possible causative pathogens. OR A patient died due to respiratory failure with VIM-PA in respiratory specimens.
Not related	The patient did not die due to infection. AND/OR The patient was merely colonized with VIM-PA. Colonization was defined as the presence of positive cultures of non-sterile sites with VIM-PA without signs of infection.
Unknown	Insufficient data were available in the medical records.

Abbreviations: VIM-PA: Verona Integron-encoded Metallo-β-lactamase-positive *Pseudomonas aeruginosa*

*Definitions of sepsis are described in Additional file 1

^1^ Centers for Disease Control and Prevention. CDC/NHSN surveillance definition of healthcare-associated infection and criteria for specific types of infections in the acute care setting 2016 [updated January 2016. Available from: https://www.cdc.gov/nhsn/PDFs/pscManual/17pscNosInfDef_current.pdf]

### Bacterial isolates

Data on all specimens in which VIM-PA had been found between 1st January 2008 and 1st January 2016 were retrieved from the electronic laboratory information system of the Erasmus MC. After recognition of VIM-PA in 2008, PCR for detection of *bla*_VIM_ was introduced and had been performed on all *P. aeruginosa* isolates with intermediate or resistant susceptibility results for imipenem (MIC ≥8 or disk diffusion of < 17 mm) and tobramycin (MIC > 4) [4]. This was also done for isolates with intermediate or resistant susceptibility results for imipenem in combination with a “highly-resistant microorganism (HRMO)” profile. The definitions of HRMO were issued by the Dutch Working party on Infection Prevention (WIP) [14]. A *P. aeruginosa* isolate was classified as HRMO when resistance was measured for three or more of the following antibiotics: aminoglycosides, fluoroquinolones, ceftazidime, piperacillin and carbapenems. Cultures positive with VIM-PA included screening cultures as well as clinical cultures.

Prior to 2013, bacterial identification and antibiotic susceptibility testing was performed using VITEK®2 (Vitek AMS; bioMérieux Vitek Systems Inc., Hazelwood, MO). Thereafter, the MALDI Biotyper (Matrix Assisted Laser Desorption Ionization-Time of Flight, Bruker Corporation) was used for bacterial identification to species level; antibiotic susceptibility testing was continued using VITEK®2. Antibiotic susceptibility results were interpreted according to the European Committee on Antimicrobial Susceptibility Testing (EUCAST) [15].

### Clinical data

Patients with a VIM-PA positive culture between 1st January 2008 and 1st January 2016 from any site who died in our hospital during this period were included. Medical records were accessible and reviewed via the electronic patient system of our hospital. All living patients had a follow-up of at least 12 months. Patients were excluded if death occurred outside our hospital, since we did not have access to patient files from other hospitals. The following patient characteristics were collected: gender, date of first VIM-PA culture, age at date of first VIM-PA culture, date of admission, date of death, nosocomial infection yes/no (positive culture with VIM-PA > 48 h after admission), ward of acquisition (ward where the patient was admitted 48 h before identification of VIM-PA) and Charlson comorbidity index. We used an updated Charlson comorbidity index to classify comorbid conditions. Compared with the original Charlson index, 12 instead of 17 comorbidities were included and weights were updated [see Additional file 2] [16].

A clinical review team consisting of two residents in medical microbiology and one intensivist retrospectively reviewed all medical records, including diagnostic results, of the patients who were included in our study. The members of this clinical review team independently assessed if mortality was related to sepsis and subsequently attempted to identify the source of the infection. CDC definitions of healthcare-associated infections were used to help determine whether an infection was present [5]. They noted their results on a standardized form [see Additional file 3], with the possibility of adding remarks and patient specific information. Subsequently, inter-rater agreement was calculated. In case of disagreement, the clinical review team discussed the patient. If subsequently no agreement could be reached, an independent infectious disease specialist evaluated the case as a fourth reviewer. The aim was to reach a minimal inter-rater agreement of 80%, which corresponds to substantial agreement or almost perfect agreement [17].

### Statistical analysis

Patient characteristics and the outcome of the clinical assessment in categories of related mortality were described. The Fleiss’ kappa statistic was used to test inter-rater agreement.

## Results

We developed a clinical tool to assess a possible relationship between presence of any culture with VIM-PA and mortality among hospitalised patients (Table 1).

### Patient data analyses

**Fig. 1:**
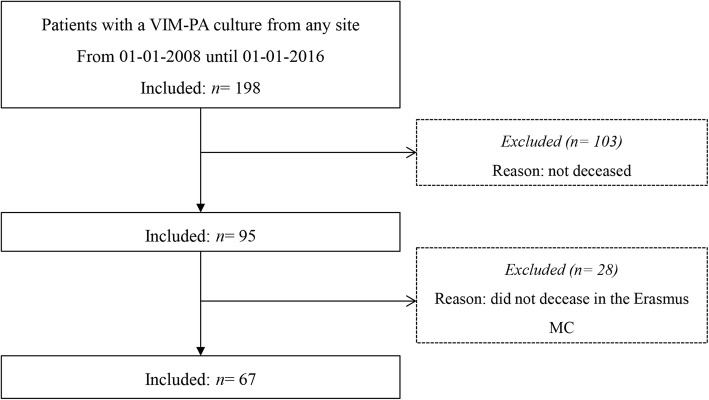
Inclusion of patients. Abbreviations: VIM-PA: Verona Integron-encoded Metallo- β- lactamase-positive *Pseudomonas aeruginosa*. Erasmus MC: Erasmus MC University Medical Center, Rotterdam, The Netherlands

A total number of 198 patients with any culture with VIM-PA between 1st January 2008 and 1st January 2016 were identified of which 95 (48%) had died at the end of the inclusion period. Of this group, 67 patients deceased in our hospital (Fig. 1). Patient characteristics are described in Table 2. The level of agreement after the first round of independent review by the clinical review team was moderate/substantial (65.7% agreement, kappa = 0.62). The level of agreement after one round of discussion was almost perfect (98.5% agreement, kappa = 0.98). For one patient, agreement could not be reached after the second round of deliberation, so an infectious diseases specialist evaluated this patient as a fourth reviewer. For another patient, all the clinical review team agreed that too little data was available in the patient files to draw conclusions. Therefore, this patient was scored as “unknown”. The patient concerned chose a restrictive medical treatment policy and died on the geriatric ward.

**Table 2:** Characteristics of included patients (*n* = 67)

Characteristic	Total	Definitely related	Probably related	Possibly related	Not related	Unknown
No. of patients (%)	67 (100)	15 (22.4)	2 (3.0)	15 (22.4)	34 (50.7)	1 (1.5)
Male gender (%)	33 (49.3)	10 (66.7)	2 (100)	7 (46.7%)	13 (38.2%)	1 (100)
Mean age at time of first isolation of VIM-PA (range, SD)	56.6 (18–82, 14.2)	53.0 (20–71, 13.3)	46.1 (40–52, 9.0)	58.3 (34–82, 12.6)	57.5 (18–80, 15.1)	79.1
Mean age at time of death (range, SD)	57.0 (18–82, 14.0)	53.3 (20–71, 13.5)	46.3 (40–53, 8.8)	58.8 (37–82, 11.8)	57.8 (18–81, 15.0)	79.1
Median LOS before first VIM-PA culture, days, (range)	23 (0–244)	22 (1–244)	85 (80–90)	37 (4–126)	22 (0–202)	0
28-day mortality (%)	36 (53.7)	11 (73.3)	0 (0)	7 (46.7)	17 (50.0)	1 (100)
1-year mortality (%)	61 (91.0)	14 (93.3)	2 (100)	14 (93.3)	30 (88.2)	1 (100)
Location of first VIM-PA; ICU (%)	49 (73.1)	13 (86.7)	0 (0)	13 (86.7)	23 (67.6)	0 (0)
Ward of acquisition; ICU (%)	47 (70.1)	13 (86.7)	0 (0)	12 (80.0)	22 (64.7)	0 (0)
Location of death; ICU (%)	43 (64.2)	14 (93.3)	1 (50.0)	9 (60.0)	19 (55.9)	0 (0)
Nosocomial acquisition	67 (100)	15 (100)	2 (100)	15 (100)	34 (100)	1 (100)
Source of infection						
Pneumonia (%)	15 (22.4)	7 (46.7)	1 (50.0)	7 (46.7)	0 (0)	0 (0)
Gastro-intestinal (%)	11 (16.4)	4 (26.7)	1 (50.0)	6 (40.0)	0 (0)	0 (0)
Pneumonia and/or gastro-intestinal (%)	2 (3.0)	1 (6.7)	0 (0)	1 (6.7)	0 (0)	0 (0)
Pneumonia and/or skin and soft tissue (%)	2 (3.0)	2 (13.3)	0 (0)	0 (0)	0 (0)	0 (0)
Gastro-intestinal and/or cardiovascular system (%)	1 (1.5)	0 (0)	0 (0)	1 (6.7)	0 (0)	0 (0)
Gastro-intestinal and/or skin and soft tissue (%)	1 (1.5)	1 (6.7)	0 (0)	0 (0)	0 (0)	0 (0)
No infection or undetermined (%)	35 (52.2)	0 (0)	0 (0)	0 (0)	34 (100)	1 (100)
Charlson comorbidity score^a^						
Median Charlson score (range)	3 (0–9)^b^	2 (0–6)^c^	0.5 (0–1)	2 (0–5)	4 (1–9)^d^	4

Abbreviations: No.; number, SD; standard deviation, ICU; intensive care unit, LOS; length of stay, VIM-PA; Verona Integron-encoded Metallo-β-lactamase (VIM)-positive *Pseudomonas aeruginosa*

^a^ Quan H, Li B, Couris CM, Fushimi K, Graham P, Hider P, et al. Updating and validating the Charlson comorbidity index and score for risk adjustment in hospital discharge abstracts using data from 6 countries. Am J Epidemiol. 2011;173(6):676–82

^b^For 2 patients data are missing

^c^For 1 patient data are missing

^d^For 1 patient data are missing

**Fig. 2:**
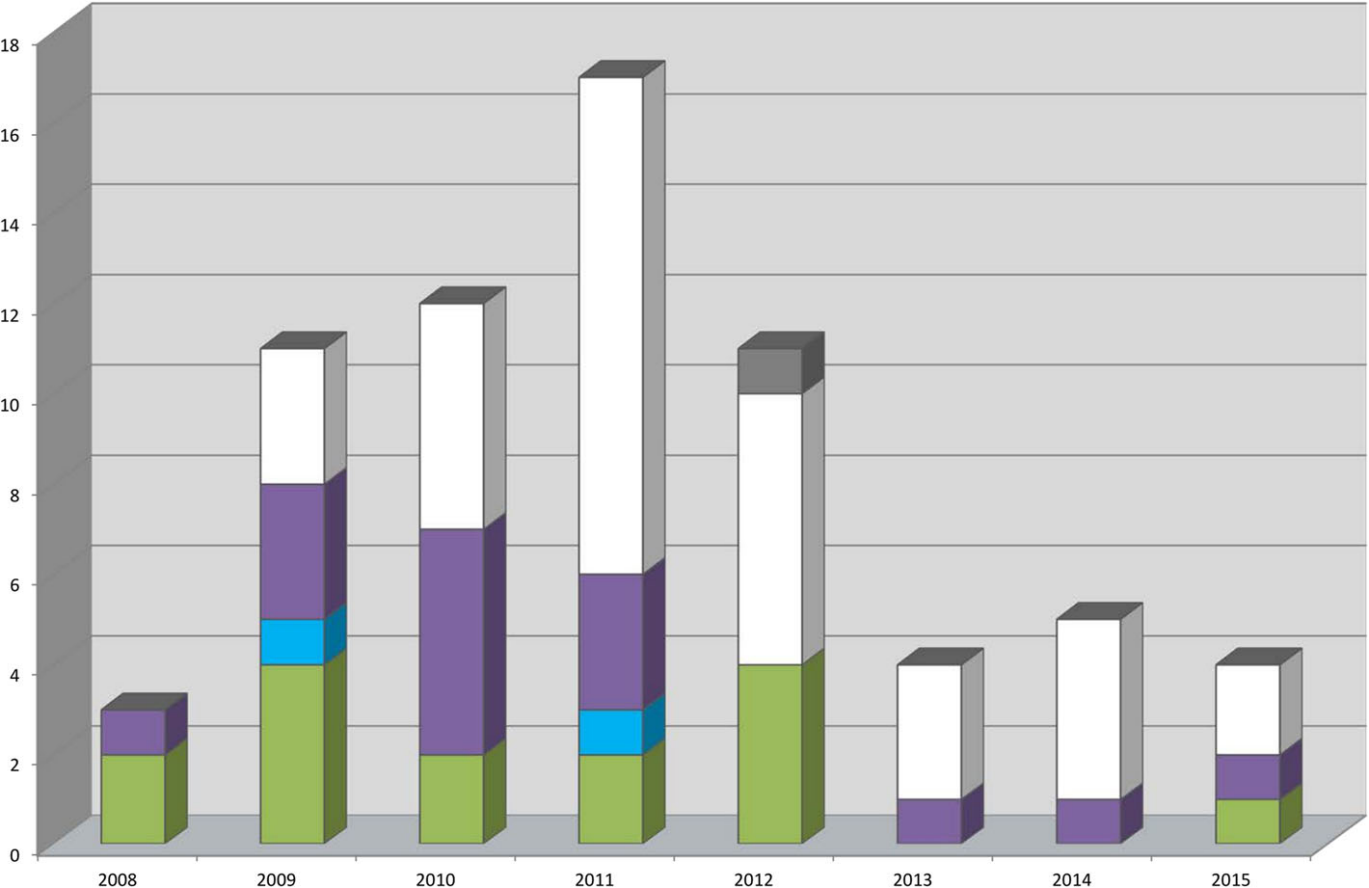
Number of patients deceased (y-axis) in each year (x-axis), n = 67 included patients. Green = definitely related, blue = probably related, purple = possibly related, white = not related, grey = unknown

The death of 15 patients (22.4%) was judged to be definitely related to an infection with VIM-PA. These patients had a recent positive blood culture with VIM-PA in combination with clinical signs of sepsis as described in the definitions of related mortality in Table 1. Furthermore, mortality of 2 (3.0%) patients was classified as probably related to VIM-PA infection and 15 (22.4%) patients were categorized in the possibly related group (Table 2). Figure 2 shows the number of patients deceased in each year, with a peak of unrelated deaths in 2011 due to the implementation of a screening programme on certain wards, leading to an increase in VIM-PA positive screening cultures.

In most patients, the bacterium was first detected at the ICU (73%) and this was the ward on which 93% of the patients of the definitely related group died. Acquisition of all VIM-PA isolates was nosocomial and the median length of stay (LOS) before the first VIM-PA culture of the 67 studied patients was detected was 23 days (range 0–244 days). On admission, the Charlson scores of the patients in the not related group were higher than in the definitely, probably and possibly related group (Charlson score ≥ 4 in 50.1, 26.7, 0 and 20% respectively, Table 2). In addition, the percentage of patients who died in the ICU was lower in the not related group compared to the definitely related group. Most infections were infections of the respiratory and gastro-intestinal tract. However, some patients had multiple sites of infection and a single source of the VIM-PA bacteraemia could not be determined (Table 2).

### Time from first VIM-PA positive culture to death

**Fig. 3:**
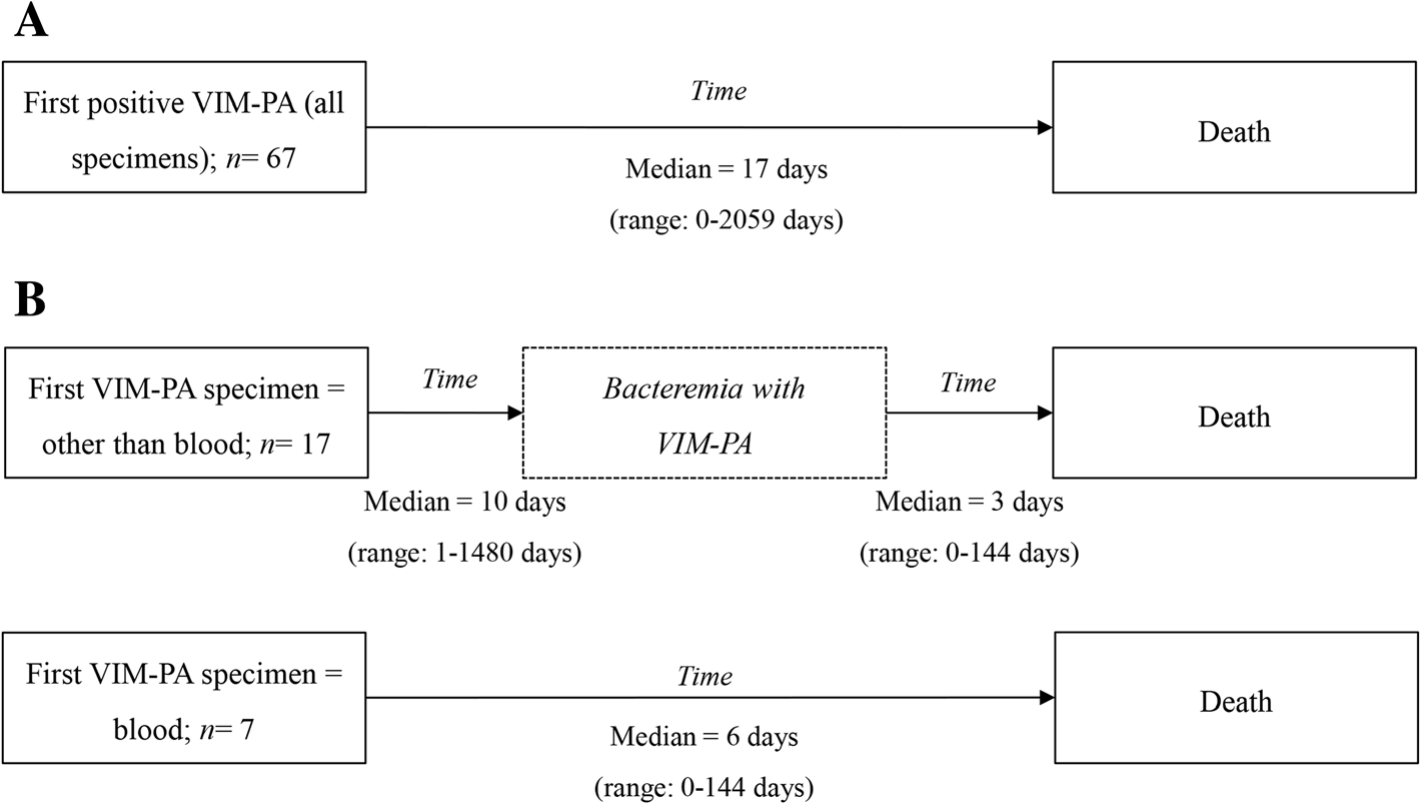
**a** Time from first detection of Verona Integron-encoded Metallo- β- lactamase-positive *Pseudomonas aeruginosa* (VIM-PA) in any specimen to death for all included patients, and (**b**) time to death in patients with a bacteraemia (i.e. as first culture or as subsequent culture)

Twenty-four out of 67 deceased patients (35.8%) had a bacteraemia with VIM-PA. Seven of these 24 patients (29.2%) had a positive blood culture as the first VIM-PA culture, of which death of five cases were considered to be definitely related to infection with VIM-PA. Median number of days from first VIM-PA positive culture to death in all included patients was 17 days compared to 6 days in patients with a positive blood culture as the first VIM-PA positive specimen (Fig. 3).

## Discussion

In this study, we developed a clinical tool consisting of a set of definitions and a scoring form for the independent review of patients’ medical charts to determine the contribution of VIM-PA to mortality. We applied this on all patients with any VIM-PA positive culture who had died in our tertiary-care hospital. Of the 198 patients with a positive VIM-PA culture in our hospital, the majority (52%) did not die during the follow-up period. Furthermore, of the patients who died in our hospital, mortality was not related to infection with this bacterium in 50.7%. However, of patients who died in our hospital (*n* = 67), the death of 15 (22%) patients was definitely related to an infection with VIM-PA. In an additional 25% of the included patients, death was probably or possibly related to an infection with VIM-PA. These results indicate that this nosocomial acquired bacterium had serious clinical impact on our patients.

The ward of acquisition in the majority of cases was the ICU, and of the definitely related group patients, all but one had deceased on the ICU. Risk factors for acquisition of VIM-PA may overlap with increased risk of dying in this patient group. In a study by Voor in ‘t holt et al., previous use of certain antibiotic regimens (e.g. use of selective digestive tract decolonisation > 10 days) and gastroscopy were identified as risk factors for VIM-PA carriage in the studied population [8].

Nosocomial pneumonia is a leading cause of mortality in patients with hospital-acquired infections and *P. aeruginosa* is one of the most important Gram-negative pathogens of hospital-acquired pneumonia (HAP) and ventilator-associated pneumonia (VAP) [18, 19]. This corresponds to our findings that the source of infection with VIM-PA was the respiratory tract in the majority of the patients (Table 2). Although most of the patients whose death was related to VIM-PA infection appeared to be ICU patients and were severely ill, this did not correspond to the Charlson scores, which is a comorbidity index on admission. The Charlson scores of the patients in the not related group were higher than in the definitely, probably and possibly related group (Table 2). We did not perform statistical analysis on these scores due to the small numbers but this implies that there were more severe comorbidities contributing to death, other than VIM-PA infection in the not related group. This is in line with admission and re-admission policies on the ICU where there is a restriction on admitting patients without any realistic survival chance, which is carefully assessed by an intensivist. The Charlson comorbidity score on admission may not be an accurate estimation of the severity of the illness at the moment of ICU admission in our study. A possible explanation could be that these patients suffered from (serious) side effects of necessary tertiary-care treatments in the hospital after admission. Once on the ICU, VIM-PA can have a clinically significant impact on mortality, also in patients with few pre-existing comorbidities on hospital admission.

Studies that evaluated the effect of antibiotic resistance of Gram-negative bacteria on mortality have produced contradicting results [9–12, 20–22]. Differences may, in part, be due to limitations of retrospective study designs, and the challenges posed to properly control for illness severity and comorbidities [23]. Although we did not quantify additional mortality due to VIM-PA in comparison to VIM-negative PA, we considered the, in this study perceived contribution of infection with VIM-PA to mortality, to be clinically relevant.

*P. aeruginosa* has the ability to form biofilms [24, 25]. This enables the bacterium to persist in the hospital environment and together with host-related factors this contributes to the susceptibility of ICU patients for nosocomial infections with *P. aeruginosa*. Breathnach et al. described two outbreaks in the United Kingdom with VIM-PA. Hospital waste water systems were found to be potential reservoirs and overall case fatality rate was 34/85 (40%) and 14/18 (78%) in patients who had bacteraemia. This corresponds to our experience with VIM-PA in our hospital where rigorous infection prevention measures were needed to control an outbreak [4, 6, 8].

Our study has several limitations. The clinical tool was developed to retrospectively assess a possible relationship between death and the presence of a VIM-PA positive culture. However, due to the complexity of most of our patients’ medical history, the causality of this relationship is not proven. Of the patients with a VIM-PA positive culture in our hospital, the majority did not die due to infection with VIM-PA within the follow-up period. Active searching for carriers can have a considerable impact on this number. Since we included only deceased patients for further analysis, we have not assessed the non-lethal complications due to possible VIM-PA infection. Of the initial 95 deceased patients in our study, 28 patients died elsewhere and were not included in our study. The true number of deaths related to VIM-PA could therefore be different but not lower than described in Table 2. The follow-up time was limited to one year for the last living patient, so possibly VIM-PA could have contributed to the death of more patients than we know of. Nevertheless, the median number of days from the first positive culture with VIM-PA to death was 17 days, so it is likely that our analysis included all relevant patients. Patients were included from several years and a peak in unrelated deaths was observed in 2011 due to the implementation of a screening programme on certain wards. On the other hand, numbers of VIM-PA related death per year remained low, making any interpretation of further analysis on correlation of results over time not reliable.

Our study results are limited to the patients who died in our hospital. The retrospective nature of this study posed limits to the available data we could review. However, we felt we had sufficient data for all but one patient to draw a conclusion on related mortality. This was due to the electronic availability of the patient files. Since most hospitals in the Netherlands work with electronic patients files, larger data sets may be analysed in the future. Currently, electronic hospital information systems differ between hospitals and data can not always be extracted in a uniform manner. Therefore, these practical challenges must be overcome before large scale analyses of mortality related to infection with (resistant) bacteria, are possible.

The results of this study may increase awareness about the presence and possible clinical consequences of a nosocomially acquired infection of VIM-PA in complex and vulnerable patients on the ICU. Furthermore, in our opinion, these results justify the extensive infection control measures which are needed to prevent spreading of VIM-PA in a healthcare facillity.

## Conclusions

We developed a clinical tool which consisted of a set of definitions of related mortality and the judgement of three physicians. With this tool, VIM-PA was shown to have an important influence on mortality in our tertiary-care patient population. Our tool may also be used for the assessment of the influence of other (resistant) bacteria on mortality, but this needs further exploration.

## Additional files

Additional file 1: Definitions and sources of sepsis. (DOCX 17 kb)

Additional file 2: Updated Charlson index score. (DOCX 14 kb)

Additional file 3: Clinical review form. (DOCX 24 kb)

## Data Availability

The datasets generated and analysed during the current study are not publicly available due to privacy regulations but are available from the corresponding author on reasonable request.

## References

[1] Blot S, Vandewoude K, Hoste E, Colardyn F (2003). Reappraisal of attributable mortality in critically ill patients with nosocomial bacteraemia involving *Pseudomonas aeruginosa*. J Hosp Infect.

[2] Kadambari S, Botgros A, Clarke P, Vergnano S, Anthony M, Chang J (2014). Characterizing the burden of invasive *Pseudomonas* infection on neonatal units in the UK between 2005 and 2011. J Hosp Infect..

[3] Osmon S, Ward S, Fraser VJ, Kollef MH (2004). Hospital mortality for patients with bacteremia due to *Staphylococcus aureus* or *Pseudomonas aeruginosa*. Chest..

[4] Van der Bij AK, Van Mansfeld R, Peirano G, Goessens WH, Severin JA, Pitout JD (2011). First outbreak of VIM-2 metallo-beta-lactamase-producing *Pseudomonas aeruginosa* in the Netherlands: microbiology, epidemiology and clinical outcomes. Int J Antimicrob Agents.

[5] Centers for Disease Control and Prevention. CDC/NHSN surveillance definition of healthcare-associated infection and criteria for specific types of infections in the acute care setting. 2016 [updated January 2016. Available from: https://www.cdc.gov/nhsn/PDFs/pscManual/17pscNosInfDef_current.pdf.

[6] Breathnach AS, Cubbon MD, Karunaharan RN, Pope CF, Planche TD (2012). Multidrug-resistant *Pseudomonas aeruginosa* outbreaks in two hospitals: association with contaminated hospital waste-water systems. J Hosp Infect..

[7] WHO. Global priority list of antibiotic-resistant bacteria to guide research, discovery, and development of new antibiotics. 2017. [updated 27–02-2017. Available from: http://www.who.int/medicines/publications/global-priority-list-antibiotic-resistant-bacteria/en/.

[8] Voor in ‘t holt AF, Severin JA, Hagenaars MBH, de Goeij I, Gommers D, Vos MC. VIM-positive *Pseudomonas aeruginosa* in a large tertiary care hospital: matched case-control studies and a network analysis. Antimicrob Resist Infect Control. 2018;7:32. 10.1186/s13756-018-0325-1 PMC5828133 29492262

[9] Hoxha A, Kärki T, Giambi C, Montano C, Sisto A, Bella A, D'Ancona F. Study Working Group. Attributable mortality of carbapenem-resistant *Klebsiella pneumoniae* infections in a prospective matched cohort study in Italy, 2012-2013. J Hosp Infect. 2016;92(1):61–6 10.1016/j.jhin.2015.06.018 26319590

[10] Falagas ME, Tansarli GS, Karageorgopoulos DE, Vardakas KZ (2014). Deaths attributable to carbapenem-resistant *Enterobacteriaceae* infections. Emerg Infect Dis.

[11] Carmeli Y, Troillet N, Karchmer AW, Samore MH (1999). Health and economic outcomes of antibiotic resistance in *Pseudomonas aeruginosa*. Arch Intern Med.

[12] Pena C, Suarez C, Gozalo M, Murillas J, Almirante B, Pomar V (2012). Prospective multicenter study of the impact of carbapenem resistance on mortality in *Pseudomonas aeruginosa* bloodstream infections. Antimicrob Agents Chemother.

[13] Bone RC, Balk RA, Cerra FB, Dellinger RP, Fein AM, Knaus WA (1992). Definitions for sepsis and organ failure and guidelines for the use of innovative therapies in sepsis. The ACCP/SCCM consensus conference committee. American College of Chest Physicians/Society of Critical Care Medicine. Chest..

[14] Kluytmans-Vandenbergh MF, Kluytmans JA, Voss A (2005). Dutch guideline for preventing nosocomial transmission of highly resistant microorganisms (HRMO). Infection.

[15] European Committee on Antimicrobial Susceptibility Testing (2017). Clinical breakpoints [Internet].

[16] Quan H, Li B, Couris CM, Fushimi K, Graham P, Hider P (2011). Updating and validating the Charlson comorbidity index and score for risk adjustment in hospital discharge abstracts using data from 6 countries. Am J Epidemiol.

[17] Hallgren KA (2012). Computing inter-rater reliability for observational data: an overview and tutorial. Tutor Quant Methods Psychol.

[18] Kalil AC, Metersky ML, Klompas M, Muscedere J, Sweeney DA, Palmer LB (2016). Management of Adults with Hospital-acquired and Ventilator-associated Pneumonia: 2016 clinical practice guidelines by the Infectious Diseases Society of America and the American Thoracic Society. Clin Infect Dis.

[19] Koulenti D, Tsigou E, Rello J (2017). Nosocomial pneumonia in 27 ICUs in Europe: perspectives from the EU-VAP/CAP study. Eur J Clin Microbiol Infect Dis.

[20] Joo EJ, Kang CI, Ha YE, Kang SJ, Park SY, Chung DR (2011). Risk factors for mortality in patients with *Pseudomonas aeruginosa* bacteremia: clinical impact of antimicrobial resistance on outcome. Microb Drug Resist.

[21] Suarez C, Pena C, Gavalda L, Tubau F, Manzur A, Dominguez MA (2010). Influence of carbapenem resistance on mortality and the dynamics of mortality in *Pseudomonas aeruginosa* bloodstream infection. Int J Infect Dis.

[22] Jamulitrat S, Arunpan P, Phainuphong P (2009). Attributable mortality of imipenem-resistant nosocomial *Acinetobacter baumannii* bloodstream infection. J Med Assoc Thail.

[23] Schumacher M, Allignol A, Beyersmann J, Binder N, Wolkewitz M (2013). Hospital-acquired infections--appropriate statistical treatment is urgently needed!. Int J Epidemiol.

[24] Fish DN, Piscitelli SC, Danziger LH (1995). Development of resistance during antimicrobial therapy: a review of antibiotic classes and patient characteristics in 173 studies. Pharmacotherapy.

[25] Mah TF, Pitts B, Pellock B, Walker GC, Stewart PS, O'Toole GA (2003). A genetic basis for *Pseudomonas aeruginosa* biofilm antibiotic resistance. Nature..

