# Supplementary material for: Mortality related to Verona Integron-encoded Metallo-β-lactamase-positive Pseudomonas aeruginosa: assessment by a novel clinical tool
Source: Antimicrob Resist Infect Control. 2019 Jun 19;8:107. doi: 10.1186/s13756-019-0556-9 (PMC6582487; doi:10.1186/s13756-019-0556-9)
Supplement: Supplementary file 1 — Definitions and sources of sepsis. (DOCX 17 kb) [file 13756_2019_556_MOESM1_ESM.docx]

Definitions of sepsis and sources of sepsis ^1, 2^.

| **Definition** | **Description** |
| --- | --- |
| Sepsis | A systemic inflammatory response syndrome (SIRS), manifested by two or more of the following conditions as a result of infection:   - Temperature <36.0 or >38.0°C, - White blood cell count <4x10^9^/l (<4000/mm^3^) or >12x10^9^/l (>12,000/mm^3^) or >10 % immature (band) forms - Heart rate >90/min - Respiratory rate >20/min or pCO2 <32 mmHg   Symptoms are part of a systemic response to the presence of an infection. Other explanations for SIRS should be explored before diagnosing sepsis. |
| Severe sepsis | Sepsis associated with organ dysfunction, hypoperfusion or hypotension. Hypoperfusion and perfusion abnormalities may include lactic acidosis, oliguria or an acute alteration in mental status. |
| Septic shock | Sepsis-induced hypotension (systolic blood pressure <90 mmHg or a reduction of ≥40 mmHg without another cause for hypotension) despite adequate fluid resuscitation along with the presence of perfusion abnormalities like lactic acidosis, oliguria or an acute alteration in mental status. |
| Primary sepsis | Sepsis with pathogenic microorganism in blood culture which is not related to infection at another site.  Clinical signs of blood stream infection (fever >38°C, and/or chills, and/or hypotension) AND intravascular device-associated bloodstream infections are classified as primary even if localized signs of infection are present at the access site. |
| Secondary sepsis | Sepsis with pathogenic microorganism in blood culture and an identical microorganism isolated from another site of infection or strong clinical evidence for another focus:   - Pneumonia - Lower respiratory infection, excluding pneumonia - Urinary tract infection - Bone and joint infection - Cardiovascular system infection - Central nervous system infection - Eye, ear, nose, throat, and mouth infection, including upper respiratory infections - Gastro-intestinal infection - Reproductive tract infection - Surgical wound infection - Skin and soft tissue infection, other than surgical wound infection - Other |

^1^ Garner JS, Jarvis WR, Emori TG, Horan TC, Hughes JM. CDC definitions for nosocomial infections, 1988. Am J Infect Control. 1988;16(3):128-40.

^2^ Bone RC, Balk RA, Cerra FB, Dellinger RP, Fein AM, Knaus WA, et al. Definitions for sepsis and organ failure and guidelines for the use of innovative therapies in sepsis. The ACCP/SCCM Consensus Conference Committee. American College of Chest Physicians/Society of Critical Care Medicine. Chest. 1992;101(6):1644-55.
