# Supplementary material for: Mortality related to Verona Integron-encoded Metallo-β-lactamase-positive Pseudomonas aeruginosa: assessment by a novel clinical tool
Source: Antimicrob Resist Infect Control. 2019 Jun 19;8:107. doi: 10.1186/s13756-019-0556-9 (PMC6582487; doi:10.1186/s13756-019-0556-9)
Supplement: Supplementary file 2 — Updated Charlson index score. (DOCX 14 kb) [file 13756_2019_556_MOESM2_ESM.docx]

Updated Charlson index score^1^

| **Variable** | **Weight** |
| --- | --- |
| Congestive heart failure | 2 |
| Dementia | 2 |
| Chronic pulmonary disease | 1 |
| Rheumatologic disease | 1 |
| Mild liver disease | 1 |
| Diabetes with chronic complications | 1 |
| Hemiplegia or paraplegia | 2 |
| Renal disease | 1 |
| Any malignancy, including leukemia and lymphoma | 2 |
| Moderate or severe liver disease | 4 |
| Metastatic solid tumor | 6 |
| AIDS/HIV | 4 |
| **Maximum comorbidity score** | **24** |

^1^ Quan H, Li B, Couris CM, Fushimi K, Graham P, Hider P, *et al.* Updating and validating the Charlson comorbidity index and score for risk adjustment in hospital discharge abstracts using data from 6 countries. American journal of epidemiology. 2011;173(6):676-82.
