# Supplementary material for: Mortality related to Verona Integron-encoded Metallo-β-lactamase-positive Pseudomonas aeruginosa: assessment by a novel clinical tool
Source: Antimicrob Resist Infect Control. 2019 Jun 19;8:107. doi: 10.1186/s13756-019-0556-9 (PMC6582487; doi:10.1186/s13756-019-0556-9)
Supplement: Supplementary file 3 — Clinical review form. (DOCX 24 kb) [file 13756_2019_556_MOESM3_ESM.docx]

**Reviewer:**

**Date:**

**FormNo:**

| **Patient characteristics** |  |  | **Remarks** |
| --- | --- | --- | --- |
| Patient name |  |  |  |
| PID |  |  |  |
| Date of birth |  |  |  |
| Gender |  |  |  |
|  |  |  |  |
|  | **Date** | **Specimen/origin** | **Other microorganism(s)? If yes specify** |
| **First positive culture with VIM-PA** |  |  |  |
| **Last blood culture(s)**  **(if BC not taken, fill in N.T)** |  |  |  |
| **Death (only date)** |  |  |  |
| **SIRS^1^ present at time of death?**  **≥2 of criteria:**  **-T<36.0 or >38.0 C**  **-WBC <4 9 109/l or >12 9 109/l or >10 % immature (band) forms**  **- heart rate >90/min**  **-resp. rate>20/min or pCO2 <32 mmHg** | **Yes/No/Unknown** |  |  |
| **SIRS caused by infection (sepsis) or is there severe sepsis/septic shock^2^?** | **Yes/ Probably/ Possibly/No/Unknown** |  |  |
| **Site of infection** | **Primary/Secondary^3^** | **If secondary please specify source^4^:** | **Specimen positive with VIM PA: Y/N**  **If Y: date and specimen:** |
| **Death related to VIM PA infection** | **DR/ PROBR/ POSR/ NR/ U** |  | **Official cause of death:** |

**Other remarks:**

1. SIRS: Systemic Inflammatory Response Syndrome

2. Definition of severe sepsis: sepsis associated with organ dysfunction, hypoperfusion or hypotension. Hypoperfusion and perfusion abnormalities may include lactic acidosis, oliguria or an acute alteration in mental status. Septic shock: is sepsis induced hypotension (systolic blood pressure <90 mmHg or a reduction of ≥40 mmHg without another cause for hypotension) despite adequate fluid resuscitation along with the presence of perfusion abnormalities like lactic acidosis, oliguria or an acute alteration in mental status.

3. Primary sepsis: pathogenic microorganism in blood culture which is not related to infection at another site. Clinical signs of blood stream infection (fever >38°C, and/or chills, and/or hypotension) AND intravascular device-associated bloodstream infections are classified as primary even if localized signs of infection are present at the access site. Secondary sepsis: pathogenic microorganism in blood culture and an identical microorganism isolated from another site of infection or strong clinical evidence for another focus.

4.Source: Pneumonia; Lower respiratory infection, excluding pneumonia; Urinary tract infection; Bone and joint infection; Cardiovascular system infection; Central nervous system infection; Eye, ear, nose, throat, and mouth infection, including upper respiratory infections; Gastro-intestinal infection; Reproductive tract infection; Surgical wound infection; Skin and soft tissue infection, other than surgical wound infection; Other

Abbreviations: BC= Blood culture; VIM PA= VIM positive Pseudomonas aeruginosa; DR= definitely related; PROBR= probably related; POSR= possibly related; NR= not related; U= unknown

| Description | Definition | | | | |
| --- | --- | --- | --- | --- | --- |
|  | Definite | Probable1 | Probable2 | Possible1 | Possible2 |
| - Cause of death = sepsis | 1 | 1 | 1 | 1 | 0 |
| - Recent (<10 days) blood culture with VIM-PA | 1 | 0 | 0 | 0 | 0 |
| - Not recent (<10 days) blood culture with VIM-PA or no culture | 0 | 1 | 0 | 0 | 0 |
| - Not recent (<1month until <10 days) blood culture with VIM-PA and no other pathogens in blood | 0 | 0 | 1 | 0 | 0 |
| - Not recent (>1 month) sterile specimen(s) with VIM-PA and no other pathogens in sterile specimens | 0 | 0 | 0 | 1 | 0 |
| - Other infection with VIM-PA according CDC definitions | 0 | 1 | 0 | 0 | 0 |
| - Strong suspicion sepsis was caused by VIM-PA | 0 | 1 | 0 | 0 | 0 |
| - Cause of death = resp. failure with resp. cultures with VIM-PA < 10 days | 0 | 0 | 0 | 0 | 1 |

Summary of definitions of related mortality (for full description see study protocol)

Abbreviations: VIM-PA; VIM-positive *Pseudomonas aeruginosa*, resp.; respiratory, CDC; centers for disease control and prevention, 0=no, 1=yes.
